# Use of Home-Based Connected Devices in Patients With Cystic Fibrosis for the Early Detection and Treatment of Pulmonary Exacerbations: Protocol for a Qualitative Study

**DOI:** 10.2196/14552

**Published:** 2021-08-18

**Authors:** Maxime Morsa, Amélie Perrin, Valérie David, Gilles Rault, Enora Le Roux, Corinne Alberti, Rémi Gagnayre, Dominique Pougheon Bertrand

**Affiliations:** 1 The Health Education and Practices Laboratory (LEPS UR 3412) Sorbonne Paris North University Bobigny France; 2 Pediatric Cystic Fibrosis Center Nantes University Hospital Nantes France; 3 Unité Mixte de Recherche 1123 Epidémiologie Clinique et EValuation Economique appliquées aux populations vulnérables Université de Paris Institut National de la Santé et de la Recherche Médicale Paris France; 4 Centre d’Investigation Clinique 1426 Unit of Clinical Epidemiology Hôpital Universitaire R Debré, Assistance Publique des Hôpitaux de Paris Institut National de la Santé et de la Recherche Médicale Paris France

**Keywords:** cystic fibrosis, pulmonary exacerbation, connected devices, patient education, self-management

## Abstract

**Background:**

Early detection of pulmonary exacerbations (PEx) in patients with cystic fibrosis (CF) is important to quickly trigger treatment and reduce respiratory damage. We hypothesized that using home-based and wearable connected devices (CDs) and educating patients to react in case of abnormal variations in a set of parameters would allow patients to detect and manage their PEx early with their care team.

**Objective:**

This qualitative study aimed to assess the feasibility and appropriate conditions of a new PEx management process from the users’ point of view by analyzing the experience of patients and of CF center teams regarding the education program, the use of CDs, and the relationship between the patient and the care team during PEx management.

**Methods:**

We have been conducting a multicenter pilot study involving 36 patients with CF aged ≥12 years. The intervention was divided into 3 phases. In phase 1 (3 months), patients were equipped with CDs, and their parameters were collected on 3 nonconsecutive days each week. Phase 2 involved the development of a “React to PEx” educational program aimed at providing patients with a personalized action plan. A training session to the educational program was organized for the physicians. Physicians then determined the patients’ personalized alert thresholds by reviewing the data collected during phase 1 and their patients’ clinical history. In phase 3 (12 months), patients were educated by the physician during a clinic visit, and their action plan for reacting in timely fashion to their PEx signs was defined. Education and action plans were revised during clinic visits. At the end of the project, the patients’ experience was collected during semistructured interviews with a researcher as part of the qualitative study. The experience of CF teams was collected during focus groups using a semistructured guide once all their patients had finished the study. The interviews and focus groups were recorded and transcribed verbatim to be analyzed. Data from educational sessions were collected throughout the educational program to be put into perspective with the learnings reported by patients. Analyses are being led by 2 researchers using NVivo (QSR International).

**Results:**

The study received the favorable reception of the Committee for the Protection of Persons (CPP NORTH WEST III) on June 10, 2017 (#2017-A00723-50). Out of the 36 patients included in phase 1, 27 were educated and entered phase 3. We completed collection of all data from the patients and care providers. Qualitative analysis will provide a better understanding of users’ experience on the conditions of data collection, how useful CDs are for detecting PEx, how useful the PEx action plan is for reacting quickly, what patients learned about PEx management, and the conditions for this PEx management to be sustainable in routine care.

**Conclusions:**

This study will open new perspectives for further research into the implementation of an optimal PEx care process in the organization of care teams in order to support patient self-management.

**Trial Registration:**

ClinicalTrials.gov NCT03304028; https://clinicaltrials.gov/ct2/show/results/NCT03304028

**International Registered Report Identifier (IRRID):**

DERR1-10.2196/14552

## Introduction

Pulmonary exacerbations (PEx) are the main cause of lung function decline in patients with cystic fibrosis (CF) leading to respiratory failure. Identifying warning signs of PEx is a priority to trigger early treatment and reduce respiratory damage [1]. Some authors have attempted to define scores based on symptoms felt and expressed by patients, particularly during telephone contact with their doctor, in order to standardize treatment [2]. However, the lack of consensus led the EuroCareCF Working Group to recommend that the medical decision regarding the prescription of an antibiotic treatment (or antibiotic modification) associated with PEx-like symptoms remains the gold standard definition of PEx in clinical trials [3].

Recently, the Standardized Treatment of Pulmonary Exacerbations (STOP) study conducted at 11 CF centers in the USA was intended to serve as a basis for future interventional studies aimed at improving the outcomes of exacerbations [4]. West et al [5] pointed out the significant heterogeneity in physicians’ decisions regarding antibiotic treatments used to treat an exacerbation. Indeed, a study testing various scenarios for designing interventions led to the conclusion that a combination of mean change in Cystic Fibrosis Respiratory Symptom Diary-​Chronic Respiratory Infection Symptom Score (CFRSD-CRISS) and in absolute forced expiratory volume in 1 second (FEV1) in liters predicted from treatment initiation should be used for performing interventional studies targeting CF exacerbations [6,7]. Whether these indicators can be used to detect and manage patients’ PEx early in routine care remains an open question. The prospect of using connected devices (CDs) to measure physiological parameters and patient perceptions at home turns this question into a matter of feasibility, reliability, and sustainability in a real-life context.

Previous studies have shown that a combination of physiological parameters and patient-reported perceptions (PRP), such as weight loss, decreased spirometry, increased cough, or increased sputum production reported daily, helps to diagnose PEx episodes and trigger early treatment [8,9]. A study aimed at establishing a consensus approach (Delphi) identified 10 signs of PEx (Tables 1-2) frequently perceived by patients and 10 indicators most often cited by caregivers [10], 4 of which were shared between professionals and patients. It was further found that professionals relied more on measurements of physiological parameters, while patients relied on perceptions and difficulties to perform their daily activities.

**Table 1:** Indicators of an exacerbation from a Delphi survey in adults with cystic fibrosis: mean scores, SD, and rank order of each statement [10]. Scores with the same average rating were given the same joint ranked position.

Statement	Score, mean (SD)	Rank order
A large decrease in lung function (greater than 10% FEV1^a^)	9.33 (0.784)	1^b^
Feeling more short of breath than usual	8.52 (1.087)	2^b^
Trouble breathing	8.52 (1.805)	2
Feeling the need to do more airway clearance than usual	8.37 (1.115)	4
An increase in symptoms at night	8.22 (1.450)	5
Producing more sputum	8.19 (1.388)	6^a^
Finding it harder than normal to do usual exercise	7.96 (1.581)	7
Finding it harder than normal to do usual activities	7.93 (1.838)	8
Feeling more exhausted than usual	7.85 (1.703)	9
More coughing than usual	7.85 (1.610)	9^a^

^a^FEV1: forced expiratory volume in 1 second.

^b^Also ranked in the top 10 by cystic fibrosis health care providers.

**Table 2:** Indicators of an exacerbation from a Delphi survey in cystic fibrosis health professionals: mean scores, SD, and rank order of each statement [10].

Statement	Score, mean (SD)	Rank order
Increased sputum	8.84 (1.027)	1^a^
A large decrease in lung function (greater than 10% FEV1^b^)	8.84 (1.263)	1^a^
More shortness of breath than usual	8.32 (1.141)	3^a^
Increased inflammatory markers (for example CRP^c^ and white cell count)	7.92 (1.124)	4
Fever or increased temperature	7.89 (1.269)	5
Increased respiratory rate at rest	7.82 (1.557)	6
Decreased oxygen saturation	7.79 (1.510)	7
Hypoxia/hypoxemia	7.76 (1.807)	8
Change in the color of sputum	7.61 (1.636)	9
New changes on chest x-ray	7.47 (1.767)	10
Increased coughing	7.47 (1.466)	10^a^

^a^Also ranked in the top 10 by adults with cystic fibrosis.

^b^FEV1: forced expiratory volume in 1 second.

^c^CRP: C-reactive protein.

At present, patients with CF do not routinely monitor their lung function at home, as few are equipped with devices to track variations in their physiological parameters or perceptions over time. Clinical observations also show that changes in physiological parameters and PRP related to PEx differ according to age and degree of lung function impairment [11]. Consequently, PEx may be diagnosed with a delay as symptoms progress while remaining unnoticed and patients seek medical care late. A few studies have been initiated in CF with daily monitoring of PEx symptoms and a few clinical parameters, mainly spirometry and oxygen saturations, together with a symptom diary [12,13]. Data were transmitted to the clinical staff who analyzed the variations and decided what course of action to take in case of an alert. These studies generally used the same alert thresholds for all patients although it is known that patients with CF have different thresholds for these indicators [9,11]. These findings have opened the way into investigating a more personalized approach to help patients self-manage their indicators at home for identifying and treating PEx early.

Our hypothesis is that an intervention that combines the provision of CDs with personalized alert thresholds and patient education by a physician may enable patients to detect early PEx signs and initiate the management of a PEx episode in a timely manner. In order for this self-management process to lead to the effective development of appropriate patient behavior, it is further hypothesized that it is essential to teach patients how identify alerts defined as abnormal variations in their parameters and to react to them.

Adults with CF report significant and unmet needs for information on the disease [14], and it has been shown that patient adherence to treatment recommendations appears to be greater when patients have a better understanding of these recommendations [15]. Shared decision-making is built on the principle that patient participation in decisions regarding their health and treatments is associated with better adherence to treatment and healthier behavior [16]. Patients therefore need to have greater control over the decisions and actions affecting their health.

In France, the CF patient education group, Groupe Éducation Thérapeutique et Mucoviscidose (GETTHEM), has been working for more than 10 years to develop patient education programs, including an educational program entitled “React to the warning signs of an exacerbation” [17]. This program aims to achieve a co-construction between patient and clinician of a clinical semiology anticipating PEx signs and to establish a shared action plan to increase self-efficacy in PEx management. Thus, recommendations regarding the optimal regimens, route and frequency of antibiotic administration, start and duration of other drug administrations, and intensification of physiotherapy at home can be discussed preventively and reassessed regularly during clinic visits.

The intervention in this study was designed by combining the use of home-based CDs and connected wearables with a patient education program derived from React—renamed React CDs—which includes alerts and personalized action plans for patients shared with their physician. Analyzing the variations in patient parameters using a cumulative sum control (CUSUM) charts may help determine and revise patient alert thresholds when needed. Engaging patients through a patient education program may result in increased awareness of PEx detection, increased commitment to treatment implementation, and thus a better ability to react early to a PEx episode. In this patient-centered approach, the goal is to achieve the most effective outcomes by integrating a better understanding of the disease into each patient’s unique experience [18]. It is a useful approach to implement new interventions based on patients’ needs using information reported from their lived experience [19].

As part of the overall research project, this qualitative study will contribute to the assessment, from the users’ point of view, of the feasibility and appropriate conditions for the use of home-based CDs by patients educated in the early detection and treatment of PEx. This assessment will be based on the experience and skills of patients or parents (of adolescents) in the self-management process of PEx, on their relationship with the care team, and on the experience and workload of the CF center teams for this protocol. The qualitative method enables us to consider the patient as a whole by exploring the patient’s subjective perceptions, beliefs, representation, or opinions of an object or a phenomenon [20,21].

Currently, we know that adherence to connected devices by patients with a chronic condition is limited: nonusage, misuse, and dropout are frequent [22]. However, we have little information regarding the use of connected devices by patients in a real-life context and the perceived barriers to their use. The qualitative study of this research project will therefore be implemented to gain in-depth understanding of how patients use connected devices in a real-life context.

If conclusive, this study may provide new prospects for further research into the optimal organization for this PEx care process to take place in the CF centers and into the evaluation of the impact on patient health and health economic outcomes.

## Methods

### Study Intervention

**Figure 1:**
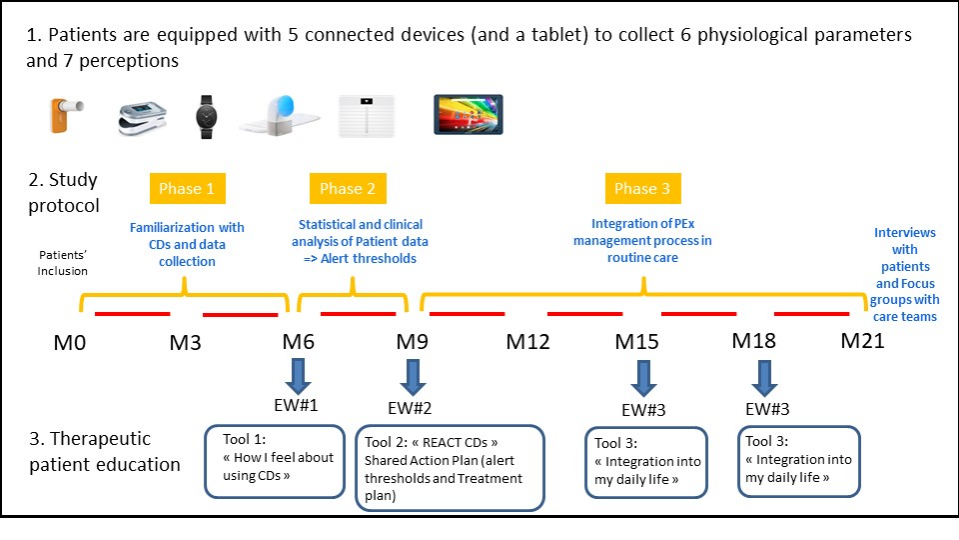
Study intervention. CD: connected device; EW: educational workshop; PEx: pulmonary exacerbations.

This 3-year pilot study was based on an intervention combining the following measures (see Figure 1).

#### Providing Patients With CDs

In 2015, a group of French CF investigators including physicians, nurses, and physiotherapists was established to select CDs capable of collecting relevant parameters with a focus on PEx detection. This group started with a literature review on parameters of interest for the detection of PEx and CF experiences on the use of CDs to collect these parameters. Five devices from two French companies (Lamirau and Withings) were selected after a market analysis conducted by a consultant specialized in telemedicine in France [23]: (1) The Oxymeter model PO3M measures blood oxygen. (2) Spirobank Smart determines the flow and volume and produces graphs to analyze the quality of the measurements. The recording of (PRPs) is proposed after the flow measurement. (3) Body cardio scale measures full body composition (weight, lean body mass) and heart rate. (4) The Activity Pop watch tracks the number of steps, distance walked, and calories burned. (5) The AURA device consists of an under-mattress sensor combined with a lamp that acts as an alarm clock. It measures the heart frequency during sleep and tracks sleep stages, the number of times the person wakes up during the night, and total sleep time.

For the study, these CDs were used to collect 13 parameters, comprising 6 physiological parameters, including FEV1, cardiac frequency, SaO2, weight, sleep duration (minutes/night), and physical activity (step count/day); and 7 PRPs, including trouble breathing, need for more airway clearance, increased symptoms at night, difficulty to perform usual activities, experience of greater fatigue, loss of appetite, and change in sputum (color or quantity).

#### Defining Personalized Alert Thresholds for Patients Through Statistical and Clinical Analysis of Patient Data

A statistical process control analysis of patient data was performed by the statistician (Institut National de la Santé et de la Recherche Médicale [INSERM]) on the data collected during the 3-month phase 1 in order to guide the physician in defining patient alert thresholds and to inform the discussion with the patient during the patient education session. CUSUM charts were then used to detect abnormal changes in physiological parameters and perceptions of each patient by comparing these variations to thresholds considered as the upper control limit or lower control limit. An automatic alert generation program was configured. In phase 3, an email was sent to the patient each time a parameter deviated from its normal limits.

#### Educating the Patient to Interpret and React to Alerts of an Acute PEx by Taking the Actions Agreed Upon With Their Clinicians to Resolve the PEx Episode.

The initial patient education program “Warning signs of a PEx in CF” was adapted by a group of expert clinicians together with a patient and a parent from a French CF patient education group (GETHEM) to take into account the patient’s alert thresholds. The new program was named “React CDs” and consisted of 3 educational workshops.

In educational workshop 1, titled “How I have felt about my daily life with connected objects since this research project started,” the patient education program started with a semistructured interview with patients conducted by the clinical research assistant (CRA) at the CF center at the end of phase 1. It was designed to gather patient feedback on this first phase and to identify patient needs and expectations regarding the next educational session. Their feedback, their overall understanding of the data collected, and their motivation to continue the study were explored using open-ended questions (Multimedia Appendix 1).

In educational workshop 2, titled “Symptoms, parameters, and action plan”, the patient education session was organized during the next clinic visit involving the CRA and the physician with the patient (or with the parent when the patient was under 18 years of age) using the React CD tool (Multimedia Appendix 2). It began by reviewing the signs and variations in the patient’s PEx for each parameter as collected from the observations made during phase 1. Next, the patient’s understanding of the course of a PEx and of the necessary gradual actions to be undertaken were explored together with the physician, as well as the barriers that may prevent the patient from seeking treatment. Finally, the patient’s alert thresholds were defined, and an action plan in the event of an alert was agreed upon by the physician and the patient. The frequency of measurement with the CDs was defined according to the patient’s daily schedule. The action plan specified gradual actions, such as intensifying physiotherapy, increasing hydration, taking additional lung clearance medication, starting oral or nebulizer antibiotics prescribed conditionally in case of PEx calling the center to make an appointment, and so on. This educational session was planned to last approximately 45 minutes with each patient.

In educational workshop 3, patient education was reviewed after 6 months, mid–phase 3, and during subsequent clinic visits, based on what had happened and whether the patient had reacted effectively. The action plan could be revised to take into account any changes in the patient’s lifestyle or health status.

### Qualitative Study Objectives

As part of the overall project, the qualitative study aims are to assess the feasibility of this PEx management process as routine care from the user’s point of view. As a pilot study, the objective is to identify the utmost difficulties expressed by users on every topic important to them or related to the context that could prevent or limit the subsequent adoption of this process in their routine care.

The context included technical aspects using CDs or internet access, patient lifestyle, accessibility to the CF care team, and health complications that may arise during the course of the study. The following themes were explored during the interviews: the acceptance of both the CDs and measurement workload for the patient, the relationship with the care team during a PEx episode, what patients learn about PEx, healthy behaviors, and the implementation of timely treatments.

In order to improve our understanding of the feasibility and conditions of this process in routine care, 2 researchers (MM and DP) will analyze the patients’ feedback at the end of phase 1 and 3, together with the documents produced during the patients’ educational sessions with the physicians, the transcripts of patient or parent interviews, and the documents from the focus groups with the clinical teams at the end of phase 3.

### Study Population

#### Sample Size

For this pilot study, the number of participants was based on the recruitment capacities of the centers, the logistical constraints, and the possibility of observing a saturation phenomenon on the qualitative study. The saturation point in qualitative studies is usually reached between 20 and 30 interviews [24]. This sample is increased by 20% to cover the risk of patients dropping out during the study. The population at inclusion was set at 36 patients.

#### Eligibility Criteria

The study population was defined to include various profiles in nontransplanted patients, adolescents or adults, living in different regions and followed in different CF centers, all with a pulmonary function status not suggesting that a transplant could be required during the course of the study (FEV1% > 50% at inclusion).

The participant inclusion criteria were as follows: 12 years of age or older, in a clinically stable condition (no PEx requiring intravenous antibiotics within the past 4 weeks), with at least 1 PEx within the past 12 months, currently being followed in a participant CF center (and not planning to change centers during the course of the study), no history of having undergone solid organ transplants, prescribed at least 1 pulmonary medication (eg, inhaled mucolytic, inhaled or oral antibiotic therapy, hypertonic saline), French speaking, able to connect a tablet to Wi-Fi, and having signed written informed consent.

Patients were deemed ineligible if they wished to participate in another therapeutic study planned at the center.

### Study Setting

#### Multicenter Study

Patients were recruited in 7 centers from 4 different geographical areas: 3 pediatric centers (4 patients per center) and 4 adult centers (6 patients per center; Table 3). These centers offer various contexts regarding the social situation of patients, for instance, if they are city dwellers or more rural dwellers, or if they live close to or far from their center.

#### Recruitment Process

Clinic staff emailed all potentially eligible patients or parents (in pediatric settings) to present them the study and offer the opportunity to opt out. Patients or parents who opted out of the study were asked to complete a questionnaire anonymously about the reasons for opting out and provided demographic data. The items were inspired by previous publications from the Pew Internet Research Center and previous research on patients with CF [25]. This questionnaire was also used for patients who dropped out of the study.

Study staff then phoned patients or parents who wished to participate and optionally patients or parents who had not opted out. They checked their eligibility, gave them information, answered their questions, and asked for verbal informed consent. The inclusion visit was then scheduled for the next clinic visit.

### Participant Timeline for the Study

#### M0: Inclusion Visit

Upon inclusion, a written informed consent form was signed by all adults or parents of adolescents; an information letter was given to the children. Quality of life and anxiety-depression scores were collected using the Health Anxiety Depression Scale (HADS) and Cystic Fibrosis Questionnaire-Revised (CFQ-R) scale. Each patient was given the 5 CDs and a tablet dedicated to the research. The CDs were synchronized with the patient’s tablet, and the necessary apps were downloaded via an anonymous ID by the CRA. Patients received a demonstration and written instructions on how to use the CDs (including cleaning and disinfection) and a maintenance support number (hotline).

#### M0-M3: Data Collection for 3 Months (Phase 1)

Patients were asked to use their CDs at home on 3 nonconsecutive days each week and to synchronize them with their tablet at least once a week. During the M3 clinic visit, quality of life and anxiety-depression data were collected using the HADS and CFQ-R scale. The number and date of diagnosis of acute PEx, FEV1, weight, respiratory symptoms, and antibiotic treatments prescribed were collected from the electronic patient record and transferred into the e-clinical research file.

#### M4 to M9: CUSUM Analysis and Education Program Setup (Phase 2)

During the clinic visit (M4-M5), patient feedback on the first phase was collected by the CRA (educational workshop 1). The ensuing educational session took place (educational workshop 2) during the next clinic visit (M9). Quality of life and anxiety-depression data were collected using the HADS and CFQ-R scale.

#### M9-M21: Data Collection and PEx Management for 12 months (Phase 3)

During the third phase, physiological parameters and PRPs were continuously collected by CDs. In the event of an alert, patients were automatically notified by email. Patients attended their clinic visits as usual. During clinic visits, their action plan was reviewed with their physician. Adherence to CD usage was measured from the audit trail that recorded each time a device was used. The number of acute PEx and the time between 2 acute PEx, FEV1, weight, respiratory symptoms, and antibiotic treatments prescribed during the period were collected in the e-clinical research file at the CF Centre.

#### Final Clinic Visit (M21)

Quality of life and anxiety-depression data were collected using the HADS and CFQ-R scale. Patients kept the CDs at the end of phase 3. Patient feedback on phase 3 was collected by the CRA (therapeutic patient education #3). Within 3 months, the final interview was conducted individually over the phone by a researcher to discuss the patients’ overall experience of this PEx management process.

### Qualitative Data Collection

There were 2 sources of data for the qualitative study. One was the data collected from patients and professionals by the CRA during the educational program with the physician; the documents completed by the patient with the CRA were transferred to the research team in charge of the qualitative analysis and stored in a secured environment. The other was data collected during patient or parent interviews and focus groups with the care teams at the end of phase 3; all interviews were recorded and transcribed verbatim for content analysis.

Patients’ experience was collected in semistructured interviews using an 8-item open-ended question interview guide (Textbox 1) derived and adapted from validated protocols for patient narrative elicitation in outpatient care experiences [20].

The experience and workload of care teams were explored in focus groups using a 5-item open-ended interview guide (Textbox 2).

**Guide for semistructured interviews with patients or parents.**

1. For you, what are the most important aspects in the management of your respiratory exacerbations in your daily life?
2. How do you rate the conditions for managing exacerbations during the study (based on what is most important to you)?
3. Can you tell us about a positive experience you had during this study concerning the management of your exacerbations? What happened and how did it make you feel? Did you do anything in particular after this positive experience (eg, change your attitude or behavior)?
4. Can you tell us about an experience that turned out differently than you expected? What happened and how did you feel at the time?
5. Regarding this last experience where you wished things had turned out differently, did you or your doctor do anything to rectify the situation?
6. Did your participation in the study change your outlook on the way you manage your exacerbations?
7. What do you think would be the best way to integrate this type of long-term follow-up so that it addresses the aspects that are most important to you in the management of your exacerbations?
8. Is there anything else you wish to tell us about (eg, COVID 19)?

**Guide for the focus group with care teams.**

1. From the point of view of the health care team, what are the most important aspects in the management of respiratory exacerbations in patients, particularly in their daily lives?
2. In your opinion, how have the proposed monitoring methods, including connected objects and patient education, addressed these priorities, or within what limits?
3. During this research project, what changes have you noticed in the way the team works or in its workload with regard to monitoring patients for the management of their exacerbations? Have you noticed a change in your relationship with the patients’ physiotherapist in town?
4. What difficulties or bad experiences have you had in the process of managing patient exacerbations using connected objects?
5. Do you feel that you had positive experiences during this study with the management of patient exacerbations? How would you rate these experiences in relation to the most important aspects of the management of respiratory exacerbations?
6. In your opinion, should this type of long-term patient follow-up be included in the management of exacerbations or in other aspects of their management? If so, what would be the best way to integrate it, and for which patients and with which objectives?.
7. Is there anything else you wish to tell us about (eg, COVID-19)?

### Qualitative Analysis of Data Collected With Patients or Parents

All interviews have been transcribed verbatim and are being subjected to a descriptive qualitative analysis. The research team will collaborate early on in the process to develop a preliminary coding framework that will be modified to incorporate additional emerging content until saturation of data is evident [24]. A grounded dimensional analysis of the patient or parent data will be performed by 2 researchers, taking into account their evolution over the course of the study and the various natures and production conditions of the collected material while constantly comparing the data within and across patients and parents [26].

### Qualitative Analysis of Data Collected With Care Teams

The data from the focus groups will be exploited (coding, categorization), processed (analysis, validity), and interpreted according to the standard thematic content analysis protocol [27]. The categories resulting from the care teams will be put into perspective within the conceptual model derived from the patients’ verbatim analysis in order to identify similar topics and specificities expressed by both categories of participants.

### Implementation of the Study

#### Training the Educators and Interviewers

The CRAs were trained in the use of the CDs and taught how to set them up for each patient included in the project. They were supported by the companies’ (Withings and Lamirau) maintenance services and hotlines in order to resolve any technical problems with the patients’ equipment.

The CRAs and the physicians who were already trained in the methodology of patient education were trained in the entire “REACT with CDs and alerts” educational program in a 1-day session for pediatric teams and a 1-day session for adult teams. The CRAs tested the interview guide in a simulated interview with a patientlike participant during the training session.

#### Process Evaluation and Monitoring

The implementation of the protocol was carried out by representatives of the promoter (INSERM). The monitoring visits were carried out by the CRA from the promoter according to the procedures and the level of risk that had been attributed to this protocol. All CF centers were monitored. At the end of the study, a monitoring and closing visit was carried out. At the end of each visit, a report was written by the CRA. Quality control procedures are described in detail in the research monitoring plan.

### Ethics Approval and Consent to Participate

Before carrying out this research, the promoter submitted the project to evaluation by a Committee for the Protection of Persons designated randomly under conditions provided for in the Public Health Code (Article L. 1123-14). Free and informed consent was collected before any act related to research was undertaken.

This research is being carried out in accordance with the reference methodology MR 001 approved by the National Commission for Computing and Liberties on July 21, 2016, and with which INSERM is committed to comply (receipt #1764311 v. 0 on January 16, 2017).

## Results

### Ethics and Approval

The whole study, including the quantitative and qualitative research, received the favorable reception of the Committee for the Protection of Persons (CPP NORTH WEST III) on June 10, 2017 (#2017-A00723-50).

### Funding

Funding for this study is from 2 main sources: Fondation pour la Recherche Médicale (FRM), who provided €173,970 (US $205,925), and Grant Vertex Pharmaceuticals, who provided €12,105 (US $14328). The Nokia Foundation (Withings) donated the CDs used for the study.

### Inclusions

In all, 36 patients have been included: 14 are children and 22 are adult patients. By the end of phase 1, 12 dropped out (5 children and 7 adults), 6 of whom participated in educational workshop 1. Finally, 24 were educated with the React CD tool (educational workshop 2) and entered phase 3 (9 children and 15 adults). Figures by centers are presented in Table 3.

**Table 3:** Number of patients involved in the study.

CF^a^ center investigator			Patients included (n=36), n		Patients educated (n=24), n
**Pediatric centers**					
	Versailles	4		3	
	Paris R. Debré	5		2	
	Nantes	5		4	
**Adult centers**					
	Lille	8		4	
	Nantes	6		6	
	Reims	3		3	
	Roscoff	5		2	

^a^CF: cystic fibrosis.

### Expected Benefits for the Participants

At the end of the study, we expect to observe new learnings in patients or parents regarding the physiological parameters impacted by a PEx, a more consistent perception of variations in these parameters [10,28], and a better understanding of the importance of early treatment to prevent degradation and possibly avoid intravenous (IV) intervention or hospitalization.

We expect to see an increased interest for patient education within the care teams despite the time-consuming nature of this activity and a stronger therapeutic collaboration between physician and patient leading to faster initiation of treatments. We intend to show that this process of care for patients equipped with CDs at home is acceptable in their daily workload or to identify the conditions necessary to make it acceptable. We hope to assess the teams’ level of satisfaction regarding the PEx action plan shared with the patients, its implementation by the patients in the event of an alert, and the positive evolution in the patients’ quality of life.

We hope to see an improvement in the evolution in PEx treatments, with lower antibiotic IV interventions for oral or nebulized antibiotics, as early diagnosis is known to allow a better recovery of previous lung function [29]. This would also result in reduced hospitalization costs, as IV cures are often initiated in hospital. We cannot anticipate the evolution of the number of PEx detected for a patient or the change in time interval between 2 successive PEx.

Qualitative analysis will provide a better understanding of the subjective experience of using such devices in a real-life context. It will allow us to identify the benefits and pitfalls of using CDs and alerts at home to detect PEx and react early, as well as the impact on the partnership between the patient and their care team.

## Discussion

### Innovation in CF Care Delivery

The use of home-based CDs is rapidly growing, and their clinical contribution to the diagnosis and resolution of PEx in patients with CF, as well as their acceptance by users deserves to be fully evaluated. The intervention in this study was designed by combining the use of home-based CDs and connected wearables with a patient education program which includes alerts and personalized action plans shared with their physician. Engaging patients through a patient education program may result in increased awareness of PEx detection, strengthened commitment to treatment implementation, and thus an improved ability to react early to a PEx episode. In this patient-centered approach, the goal is to achieve the most effective outcomes by integrating a better understanding of the disease into each patient’s unique experience [18]. This approach differs from other studies in which alerts are used by the care teams to drive actions, as our intervention focuses on ensuring that patients initiate actions themselves when alerted.

### Patient Education and Partnership With the Care Team

Patient education is a critical component of chronic care and is recognized to improve self-management. All investigative centers in France have been involved in the care quality improvement program deployed since 2011 [30]. This program has promoted patient education as an integral part of CF care to improve not only the care provided at the center but also self-care at home. Despite this involvement, not all patients have been educated to “React to signs of an exacerbation.” The educational part of this protocol will promote access to this education in the context of the use of CDs and will engage physicians with their patients as an additional benefit of the study.

Nevertheless, if patients do not follow the action plan agreed upon with their physician, their treatment of PEx may not begin earlier. An alternate process could then be envisaged to increase effectiveness, in which alerts are sent to the clinical teams and used by them to drive actions with the patient. In routine care, the process leader may switch from the patient to the care team at certain critical times when the patient’s condition worsens.

### Perspective for a CF-Integrated eHealth Solution

The use of several CDs from 2 different companies (Lamirau and Withings) led to the combination of 2 different systems for the extraction and transmission of data to the research server at INSERM. The return transmission of alerts to the patients was conducted via an email account set up for the research. This configuration does not allow the patient to use a single dashboard to gather their history of physiological parameters and PRPs or information on their treatment over the different periods. The development of a CF application that can display patient data collected from various CDs in a single dashboard is becoming increasingly necessary due to the continual emergence of new and more efficient CDs capable of measuring lung function parameters, as well as nutritional status and glycemia, which is a comorbidity in approximately 30% patients with CF and that should be part of the CF patient follow-up [31].

### Conceptualization of a Model for Health Behavior Adoption During an eHealth Intervention

Previous studies have highlighted that the use of information technology depends on its perceived usefulness and perceived ease of use [32], as well as on personal (age, gender, and previous experience) and contextual factors, including facilitating conditions, social influence, hedonic motivations, and price value [33]. The adoption of wearable devices for health self-quantification also involves “task-technology fit” characteristics, such as connectivity and healthcare infotainment, as well as a good level of perceived data privacy [25,34]. During the qualitative analysis, the researchers refer to these theoretical models when eliciting verbatim test categories from patients, which may include the perceived ease of use of CDs and alerts to manage PEx, the perceived role of education to feel at ease with the use of CDs and alerts, the perceived usefulness of gaining better control over one’s own health, or the perceived usefulness of linking one’s own perceptions to the measures given by CDs and possibly developing the ability to anticipate crises and manage them independently. Moreover, in the field of chronic care, the relationship with the care team will be addressed as an element of the model. Certain conditions facilitating or hindering the use of technology related to technical problems, the very design of the intervention, or events in the patient’s life or health will be explored. A conceptual model will be proposed for the design of interventional research on eHealth related to the phenomenon under study.

### Limits and Measures Taken

Our feasibility study includes a small sample of patients from several CF centers, which will result in a variety of situations and cases with no statistical weight. The patients included were selected on the basis of their motivation to participate in the study as solicited during the recruitment process. The small number of patients included in this pilot study will not make it possible to specify the characteristics of patients best suited to this care process using CDs at home.

Technical difficulties in the usage of CDs or with internet connections at home, depending on where the patient lives and the maturity of the device, have discouraged patients from collecting their data as regularly as needed. We had planned to recruit 36 patients at inclusion and expected 30 patients to remain until the end of the study. This expectation proved to be overly optimistic due to the technical problems encountered with the selected devices, especially regarding spirometry.

### Perspective for Further Research

This pilot study will help to define the conditions for a further trial aimed at evaluating the potential generalization in the organization of care teams and the cost-effectiveness of this care process on patient health outcomes and hospital costs in terms of the number of clinic visits, hospitalizations, and patient transportation costs.

## Appendix

Multimedia Appendix 1 React CDs: How I feel about my daily life with connected devices.

Multimedia Appendix 2 React CD's symptom parameters action plan.

## Abbreviations

CDs: connected device
CF: cystic fibrosis
CFQ-R: Cystic Fibrosis Questionnaire-Revised
CFRSD-CRISS: Cystic Fibrosis Respiratory Symptom Diary-Chronic Respiratory Infection Symptom Score
CPP: Committee of Protection of Persons
CRA: clinical research assistant
CUSUM: cumulative sum control
FEV1: forced expiratory volume in 1 second (liters)
FRM: Fondation Pour la Recherche Médicale
GETTHEM: Groupe Éducation Thérapeutique et Mucoviscidose
HADS: Health Anxiety Depression Scale
INSERM: Institut National de la Santé et de la Recherche Médicale
IV: intravenousˍ
PEx: pulmonary exacerbation
PRP: patient-reported perceptions
STOP: Standardized Treatment of Pulmonary Exacerbations
