# Supplementary material for: Use of Home-Based Connected Devices in Patients With Cystic Fibrosis for the Early Detection and Treatment of Pulmonary Exacerbations: Protocol for a Qualitative Study
Source: JMIR Res Protoc. 2021 Aug 18;10(8):e14552. doi: 10.2196/14552 (PMC8411325; doi:10.2196/14552)
Supplement: Multimedia Appendix 1 [file resprot_v10i8e14552_app1.doc]

MUCOEXOCET « REACT with Connected Devices »

Interview Guide

Step 1: *How I feel about my daily life with connected devices since this research project started.*

Today, we’ll focus on how you feel about using connected devices in your daily life and particularly on the measurements regarding your health in the 3 months of this research project. I am going to take notes during this conversation, which will help us, as professionals, to prepare the next educational session and adapt it to your needs and objectives.

**1/ How did this first phase go in your daily life?** (technical aspects of the devices to collect the data – internet, Bluetooth, devices…)

- Frequency of measurements
- Practical conditions
- Connected devices
- Moments and duration of these measurements
- Where did you do them (bathroom, kitchen…)?
- If you changed location (holidays, weekend away, alternating custody…), did you take the devices with you? Which one did you take?

**2/ Did you read the emails sent to the private email address dedicated to the research project during this period? ⃝ yes ⃝ no**

- Why?

**3/ Did you read the measures displayed on the screen of the connected devices?**

**⃝ yes ⃝ no**

- If yes, which measures? Every time you took them?
- Did they change from the beginning to the end of the 3-months period?

**4/ What observations did you make about these measures?**

**5/ Regarding the results of the measurements:**

- - What did you learn and understand?
- What surprised you? What did you think about the measures?
- Did these observations evolve over time? In what sense?
  - How did you feel about these data? (emotions, fears, confidence, self-assurance…)

**6/ Do you know which parameter is transmitted by which device?**

| Parameter | Device |
| --- | --- |
|  | Weight scale |
|  | Watch |
|  | Sleep |
|  | Spirometer |
|  | Saturometer |

**7/ How would you define an exacerbation? What does this word mean for you?**

- - Do you think these measures could change during an exacerbation? How so? Do you know why?

| Parameter | Modification  yes / no | Increase/decrease | Why? What is the reason for the change? |
| --- | --- | --- | --- |
| FEV1 |  |  |  |
| Saturation |  |  |  |
| Weight |  |  |  |
| Cardiac frequency |  |  |  |
| Hours of sleep |  |  |  |
| Step count |  |  |  |

## 8/ Following this experience, what are your questions? What explanations do you need?

**9/ In a few weeks’ time, you will benefit from an educational session with your doctor and myself (nurse, research assistant) to discuss and agree on the best way to use the connected devices in your daily life and how to react to early signs of exacerbations.**

**What are your expectations for this educational session?**

**10/ During this session, we’ll make sure to clarify together which perceptions can be signs of exacerbations, how the data measured with the connected devices can vary during an exacerbation, and which alert thresholds can be determined in your particular case, to implement actions to prevent aggravation.**

**Alerts will be sent to your email address in case measures go beyond the alert thresholds.**

**What are your preferences to schedule these alerts (morning, evening…)?**

**11/ Can you tell me how you feel about the protocol? Fearful, hopeful, perplexed, determined, motivated, regretful…**

### 12/ To what extend do you think the use of connected devices helps you to control your health better?

### (from 0: no help, to 10: great help)

**
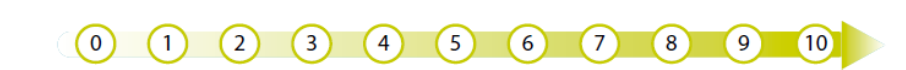
**

Could you tell us why?

### To what extent do you think the use of connected devices changes your relationship with your care team? (from 0: no change, to 10: significant change)

###

Could you tell us in which why?

### To what extent do you think the use of connected devices has been a constraint in your daily life? (from 0: no constraint, to 10: major constraint)

### To what extent do you think the use of connected devices has helped you in your daily life? (from 0: no help, to 10: great help)

**8/ Is there anything else you would like to tell us about?**
