# Supplementary material for: Use of Home-Based Connected Devices in Patients With Cystic Fibrosis for the Early Detection and Treatment of Pulmonary Exacerbations: Protocol for a Qualitative Study
Source: JMIR Res Protoc. 2021 Aug 18;10(8):e14552. doi: 10.2196/14552 (PMC8411325; doi:10.2196/14552)
Supplement: Multimedia Appendix 2 [file resprot_v10i8e14552_app2.doc]

| **Patient #:**  **Date:** | | **Parameters when my respiratory status is stable** | | | | **When I am deteriorating** | | | | **When I am really deteriorating** | | |
| --- | --- | --- | --- | --- | --- | --- | --- | --- | --- | --- | --- | --- |
|  |  | Signs / perceptions/ ☺ in Journal CDs | | | What I could improve | Signs / perceptions  ☹ in CDs journal | | What I do | What I could improve | Signs / perceptions  ☹ in CDs journal | What I do | What I could improve |
| My Perceptions | My secretions are: | ☺ | | |  | ☹ | |  |  |  |  |  |
|  | My cough is: |  | | |  |  | |  |  |  |  |  |
|  | My breathing is: | ☺ | | |  | ☹ | |  |  |  |  |  |
|  | My appetite is: | ☺ | | |  |  | |  |  |  |  |  |
|  | My sleep is:  Symptoms at night: | ☺ | | |  |  | |  |  |  |  |  |
|  | Levels of fatigue: | ☺ | | |  |  | |  |  |  |  |  |
| My activities | Physical activities: |  | | What benefit do  I get from it  from 1 to 5 |  |  | |  |  |  |  |  |
|  |  |  |  |  |  |  |  |  |  |  |  |  |
|  | Sport activities - hours/week: |  | | What benefit do  I get from it  from 1 to 5 |  |  | |  |  |  |  |  |
|  |  |  |  |  |  |  |  |  |  |  |  |  |
|  | Number of floors I can climb without shortness of breath: |  | | |  |  | |  |  |  |  |  |
|  | My social life is: | ☺ | | |  |  | |  |  |  |  |  |
| Data from my connected devices |  | Value of parameters | # of measures / week | How useful  from 1 to 5 |  | ALERT THRESHOLDS D’ALERTE |  |  |  |  |  |  |
|  | FEV1 |  |  |  |  |  |  |  |  |  |  |  |
|  | SaO2 |  |  |  |  |  |  |  |  |  |  |  |
|  | Weight |  |  |  |  |  |  |  |  |  |  |  |
|  | Resting cardiac frequency |  |  |  |  |  |  |  |  |  |  |  |
|  | Average number of hours of sleep  per night |  |  |  |  |  |  |  |  |  |  |  |
|  | Average number of steps per day |  |  |  |  |  |  |  |  |  |  |  |
| Treatments (cf. prescription) |  | Treatment | # of time/day | How useful  from 1 to 5 |  |  | |  |  |  |  |  |
|  | I drink about (liters/day):  I take additional salt up to (cp/day): |  |  |  |  |  | |  |  |  |  |  |
|  | Physiotherapy …/week  + self-drainage …/week | ☺ |  |  |  | ☹ | |  |  |  |  |  |
|  | Inhaled antibiotics: |  |  |  |  |  | |  |  |  |  |  |
|  | Bronchodilators: |  |  |  |  |  | |  |  |  |  |  |
|  | Inhaled corticoids: |  |  |  |  |  | |  |  |  |  |  |
|  | Other respiratory treatments: |  |  |  |  |  | |  |  |  |  |  |
